# Smart materials: innovative strategies for oral-maxillofacial bone defects repair

**DOI:** 10.3389/fbioe.2025.1629292

**Published:** 2025-08-26

**Authors:** Yilin Yu, Zhenyuan Liu, Xu Qin, Ke Song, Lianyi Xu

**Affiliations:** ^1^ Department of Stomatology, Tongji Hospital, Tongji Medical College, Huazhong University of Science and Technology, Wuhan, China; ^2^ School of Stomatology, Tongji Medical College, Huazhong University of Science and Technology, Wuhan, China; ^3^ Hubei Province Key Laboratory of Oral and Maxillofacial Development and Regeneration, Wuhan, China

**Keywords:** smart materials, stimuli-responsive materials, bone tissue engineering, oral-maxillofacial bone, bone repair and regeneration

## Abstract

Oral-maxillofacial bone defects complicated by tumors, infections, or other bone diseases pose a significant clinical challenge. Traditional tissue-engineered bone substitute still has limitations regarding its three elements that resulting in unsatisfactory regeneration capability. Smart materials are a cutting-edge type of functional materials that can sense and respond to a wide range of environmental conditions or stimuli, including optical, electrical, magnetic, mechanical, thermal, and chemical signals. According to the type of stimulus to which the materials respond, they can be classified into externally stimulated materials and internally stimulated materials. This review, based on the latest advances in smart materials for bone defect repair, summarizes the different stimulus-responsive strategies of smart materials and the materials under each strategy. It also discusses the classic biomedical applications of these materials in the repair of oral-maxillofacial bone injuries in recent studies, compares the advantages and disadvantages of different strategies, and discusses the current challenges and future prospects of smart materials.

## 1 Introduction

Bone defects in the oral and maxillofacial region are common clinical challenges, severely affecting patients’ masticatory function, facial appearance, and quality of life. During the process of repairing these bone defects, a variety of biomaterials are widely used.

Bone tissue engineering integrates biomaterial scaffolds, cells, and bioactive factors to construct biomimetic structures to enhance bone regeneration (Koons et al., 2020). The application of additive manufacturing technology and topographical, chemical, and/or biochemical modifications has continuously enhanced the osteogenic activity of bone tissue engineering (Zhang et al., 2019b; 2020). Recently, researchers gradually recognized that the repair process of bone defects is not a static and one-stage process (Li et al., 2024). Bone regeneration and remodeling are long-term dynamic processes. Therefore, there is a need to develop responsive biomaterials that can synchronize the interactions between the material and the surrounding tissues in both space and time. Meanwhile, challenge pathological conditions, such as bacterial infection, chronic inflammation, and disorders affecting systemic metabolism, raised the difficulties of local regenerative capability of the defect area, which requests more complex approaches to simultaneously cope with adverse metabolic conditions and stimulate tissue regeneration (Monfoulet et al., 2014; Tao et al., 2020).

**FIGURE 1:**
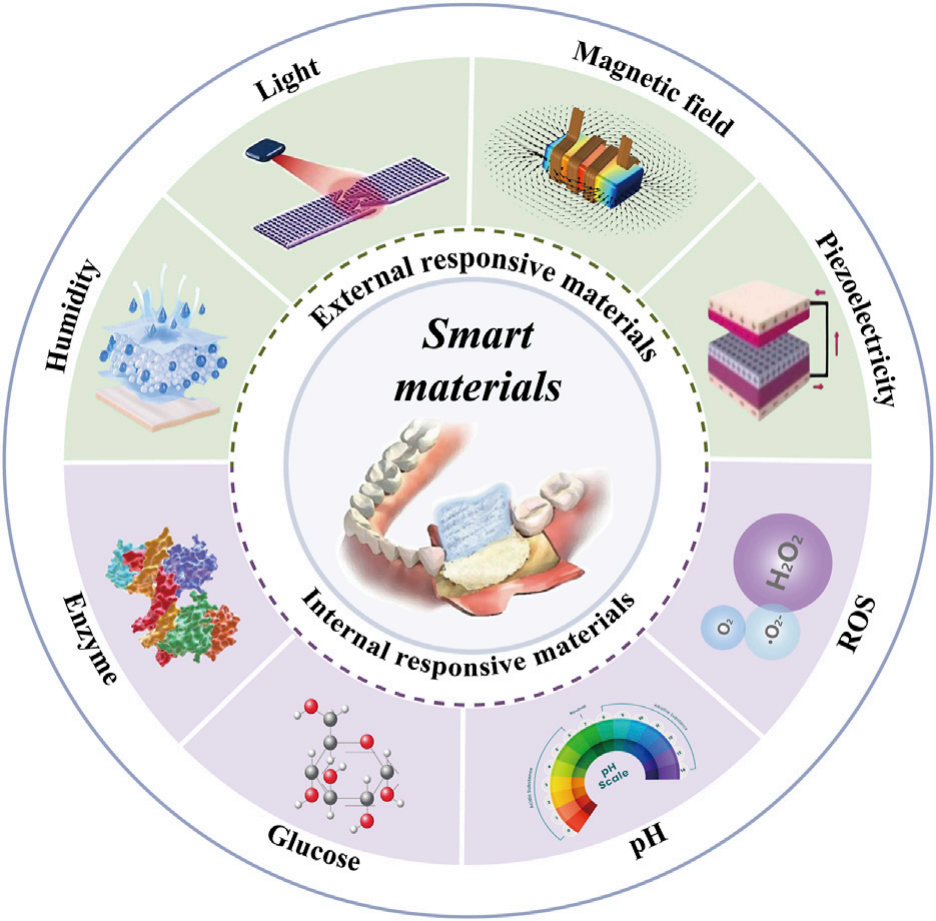
Mechanisms and Stimuli Factors of Smart Materials. Abbreviations: ROS, Reactive Oxygen Species; pH, potential of hydrogen.

In this context, smart materials, also known as responsive biomaterials, have emerged. These smart materials retain the basic framework of traditional materials but have been endowed with the ability to sense and respond to environmental changes through innovative approaches such as the introduction of functional groups, the incorporation of electromagnetic materials, the reconfiguration of material structures, and the embedding of sensors (Wei et al., 2022). This dynamic responsiveness enables smart materials to better adapt to the complex microenvironmental changes during bone defect repair, thereby enhancing the effectiveness and efficiency of tissue regeneration (Table 1). The types of stimuli that smart materials can respond to can be divided into external stimuli (such as light irradiation, electric and magnetic fields, ultrasound, and appropriate mechanical stimulation) and internal stimuli (such as excess reactive oxygen species (ROS), slight acidity, endogenous electric fields, specific ion concentrations, secreted enzymes, or specific immune environments) (Figure 1).

**TABLE 1:** Advantages of smart materials over traditional materials.

Material properties	Limitations of traditional materials	Solutions provided by smart materials	References
Bioactivity	Passive conduction, no osteo-inductivity	Controlled release of growth factors	Krishna et al. (2015), Bansal et al. (2020), Li et al. (2021)
Environmental Responsiveness	Static structure, poor adaptability to infection/mechanical environment	Defined or programmable shape changes by environmental stimuli	Lendlein and Gould (2019), Shang (2019)
Vascularization Capability	No active angiogenic capability	Integrates angiogenic functional materials and multiple biological factors	Nicosia et al. (2023), Yuan et al. (2023)
Synchronization of Material Degradation and Bone Regeneration	Uncontrollable degradation rate	Tunable material degradation for bone reconstruction	Xu et al. (2018), Lu et al. (2025)
Personalization and Biomimetic Precision	Macroscopic matching is acceptable, but microscopic structure is coarse	Custom-engineered scaffolds that closely mimic native tissue physiology	Aljohani and Desai (2018), Aversa et al. (2018)

In this review, we summarized the different stimulus-responsive strategies, including smart materials under external and internal stimulus-responsive strategies, and elaborated on the classic biomedical applications for oral and maxillofacial bone injury repair in recent studies. We also compared the advantages and disadvantages of different strategies and discussed the current challenges and future prospects of these new biomaterials. This knowledge may help to construct multifunctional biomaterials in the future to meet the needs of oral-maxillofacial bone defects repair in different environments.

## 2 External stimuli

External stimuli such as light, magnetic fields, electrical stimulation, and appropriate mechanical stimulation can generate heat or promote the adhesion, proliferation, and differentiation of osteoblasts within scaffolds, thereby facilitating bone therapy and regeneration. In this section, we elaborate on various external stimulus-responsive strategies (Table 2).

**TABLE 2:** Types of external stimuli, materials or methods, effects, and applications.

Stimulus	Materials or methods	Effects	Application	References
Photothermal	Chemical modification or elemental doping of hydrogels/polymers/metallic compound	(1) Thermal effects of light energy conversion (2) Molecular structural changes triggered by light	Photothermal therapy, antibacterial, anti-inflammatory, and bone formation promotion	Hu et al. (2024), Zhu et al. (2024), Chen et al. (2025)
Magnetic field	Nanoparticles doped with iron compounds	Magnetic field attracts magnetic particles	(1) Programmed drug release (2) Influence on cell osteogenic activity (3) Antibacterial and anti-inflammatory effects	Long et al. (2023), Ma et al. (2023), Wu et al. (2024)
Humidity	Porous structures that can adsorb water molecules	Adsorption and desorption of water molecules by the material	Degradation and ion release, antibacterial, osteogenic, angiogenic, and nerve repair-promoting effects	Shuai et al. (2022), Yang et al. (2024)
Piezoelectricity	Nanoparticles or hydrogels loaded with piezoelectric fillers	Piezoelectric charges generated by ultrasonic vibration or direct force application	Piezoelectric effect and drug release, regulating the microenvironment of bone defects, promoting osteogenesis, angiogenesis, and anti-inflammatory effects	Roldan et al. (2023), Wu et al. (2023), Zhou et al. (2024a), Yue et al. (2025)

### 2.1 Photothermal

Photothermal stimulation refers to the process of achieving regulatory effects by converting light energy into thermal energy (Liu A. et al., 2025). The core components of photothermal responsive materials comprise: (1) Photothermal conversion components (e.g., graphene, black phosphorus, and gold nanoparticles), which efficiently absorb specific wavelengths of light, thereby inducing transformations such as material deformation, drug release, or bioactivity activation (Huang et al., 2017; Chen et al., 2019; Bisoyi and Li, 2022); (2) Bone repair matrices (e.g., polylactic acid, polycaprolactone, hydrogels, or β-tricalcium phosphate), which provide mechanical support and facilitate the delivery of osteogenic factors (such as magnesium ions) (Zhang D. et al., 2024; Liu A. et al., 2025).

Commonly utilized light sources encompass near-infrared (NIR) and ultraviolet (UV) radiation. NIR is widely utilized for photothermal effects due to its exceptional tissue penetration depth, enabling energy delivery to deep tissues. This capability is attributed to minimal absorption by hemoglobin and water molecules, ensuring limited energy attenuation during therapeutic applications (Zhang J. et al., 2024). Under photothermal stimulation, scaffolds exhibit a shape memory effect: heating above the transition temperature softens the material, allowing it to conform to irregular bone defects; subsequent cooling solidifies the scaffold into the desired geometry (Wang H. et al., 2025; 2025c). Concurrently, photothermally triggered temperature elevation induces material expansion or cleavage of chemical bonds, enabling precise release of anti-inflammatory drugs, growth factors, or other bioactive agents (Zhang D. et al., 2024). In photothermal-activated systems, mild heating (42 °C) upregulates heat shock protein HSP70 and activates the MAPK/ERK osteogenic pathway. This process further promotes the release of mineralizing ions (e.g., Ca^2+^, PO_4_^3-^), thereby accelerating tissue mineralization (Zhu et al., 2024).

The application of photothermal stimulation is highly controllable; NIR can be exactly delivered to the defect site to achieve therapeutic effect. Moreover, it is non-invasive, non-toxic, and possesses high biosecurity. The photothermal effect itself has the ability to regulate the immune microenvironment, promoting the polarization of macrophages from pro-inflammatory M1 to anti-inflammatory M2, reducing inflammation and enhancing angiogenesis (Zhang D. et al., 2024; Zhu et al., 2024). In addition, UV (wavelength 254–365 nm) can also induce the formation of a three-dimensional network structure between material molecules, serving as a stimulus for photo-responsive materials. Ding et al. developed a photo-responsive hydrogel for bone tissue formation, which is composed of a photo-cross-linkable polymer solution, a photo-initiator, and a UV absorber. Upon UV irradiation, the polymer undergoes photo-crosslinking to form a solid filler that can carry human bone marrow mesenchymal stem cells, facilitating osteogenic differentiation (Ding et al., 2022). Similarly, Hu et al. achieved the modification of hyaluronic acid with methacrylic anhydride, enabling the hyaluronic acid to undergo photo-crosslinking under UV irradiation to form a stable three-dimensional network structure. Based on this structure, an antibacterial agent was loaded, making it an injectable material with both oral-maxillofacial defect repair and antibacterial functions (Hu et al., 2024).

In addition to the encouraging achievements of the aforementioned photo-responsive biomaterials, there are still unresolved issues. NIR has low penetration efficiency in deep tissues, which affects the photothermal conversion effect in maxillofacial scaffold defects, thereby ultimately hindering the regeneration of deep tissues *in vivo* (Chen et al., 2020).

### 2.2 Magnetic field

Magnetic field (MF) is a non-invasive stimulation, which excels in high tissue penetration, less toxic side effects and high controllability. External static magnetic fields (SMFs) have direct biological effects on cells and promote osteogenesis of mesenchymal stem cells (MSCs) by affecting cell metabolism and signaling (Yan et al., 2025). The enhanced osteogenesis is thought to be associated with MF-induced opening/closing of ion channels, cytoskeleton remodeling, cellular membrane potential elevation of the stimulated osteoblasts. Also, the biological effects of MF also act on pathogens. Wu et al. reported the disruption of bacterial biofilms by integrating magnetic nanoparticles (MNPs) into tricalcium phosphate scaffolds under the action of SMFs, which led to the effective control of infection (Wu et al., 2024).

The construction of magnetic-responsive materials is usually based on MNPs, dominated by Fe_3_O_4_, Fe_2_O_3_, and FeO. Chen et al. has designed a magnetic-responsive composite coating by loading γ-Fe_2_O_3_ nanoparticles onto TiO_2_ nanoporous arrays, which promotes cell proliferation and accelerates osteogenesis under SMFs (Chen et al., 2024). Some studies have combined magnetic silica nanoparticles with MSCs to prepare magneto-mechanical-bioengineered MSCs, which can activate the YAP/β-catenin signaling pathway under SMF to promote osteogenesis, mineralization, and angiogenesis, while decease bone resorption and rebalancing bone metabolism (Yu C. et al., 2023).

In addition, magnetic materials improve the mechanical strength of scaffolds. One study significantly enhanced the mechanical properties of hydrogel by introducing Fe_3_O_4_ nanoparticles and tannic acid (Zou et al., 2023). Magnetic materials can be used for remote drug-controlled release with the help of MF. A study has developed a double crosslinked magnetic hydrogel for remote controlled pulsatile release of parathyroid hormone by MNPs, which can mimic the clinical mode of drug delivery (Long et al., 2023).

Magnetic responsive materials have significantly enhanced the functions of bone implant materials, but their biosafety issues need to be noted. Degradation of magnetic materials *in vivo* products may be cytotoxic. MNPs may release metal ions (Co^2+^, Fe^3+^) after degradation *in vivo*, and long-term accumulation can easily induce cytotoxicity or inflammatory reactions (Liu W. et al., 2023). Therefore, biodegradable magnetic phases should be developed, or the risk of ion leakage should be reduced through surface functionalization such as coating with stem cell membranes (Wu et al., 2024). On the other hand, magnetic fields have tissue penetration capabilities, energy attenuation is significant with increasing depth (Shen et al., 2023). Therefore, for deep bone defects, multi-level amplification strategies can be combined to enhance local magnetic field strength (Cedillo-Servin et al., 2024).

### 2.3 Humidity

Humidity-responsive smart materials detect environmental humidity changes and generate controllable physical or chemical responses. These responses—such as swelling, contraction, degradation, or drug release—promote bone tissue regeneration. The mechanism relies on material hydration/dehydration via water molecule adsorption/desorption, primarily driven by physical interactions (e.g., hydrogen bonding, van der Waals forces) between the material and water (Shuai et al., 2022; Du et al., 2023; Mao et al., 2024; Yang et al., 2024).

Many synthetic polymers exhibit humidity responsiveness. The molecular chains of thermoplastic polyurethane contain numerous amino (N–H) and carbonyl (C=O) groups. Upon exposure to moisture, water molecules form hydrogen bonds with these functional groups, leading to hydration-induced deformation. This property renders thermoplastic polyurethane an ideal candidate for bone defect repair in minimally invasive surgery (Zhang Y. et al., 2019). Moreover, incorporating naturally humidity-responsive polymers into synthetic polymers enhances composite properties while maintaining structural stability. For instance, amorphous calcium-magnesium pyrophosphate possesses substantial free volume and active sites, enabling rapid hydration-driven expansion. Cassava starch contains abundant hydroxyl groups that form water-absorbing hydrogen bonds. Amorphous calcium-magnesium pyrophosphate/cassava starch composite scaffolds exhibit rapid humidity response: their swelling increases porosity, promote cell/cytokine attachment, while the expansion rate matches bone growth, thereby supporting *in vivo* tissue regeneration (Yang et al., 2024). Similarly, silk fibroin protein extracted from silkworm cocoons was applied. Silk fibroin comprises disordered hydrophilic (amorphous) regions and crystallizable hydrophobic blocks (β-crystal regions). The water-soluble hydrophilic regions confer elasticity and toughness, enabling hydration-driven shape memory. MgO particles were incorporated to modulate degradation rate, enhancing the material’s adaptability to *in vivo* bone tissue regeneration (Mao et al., 2024).

Humidity-responsive materials hold significant potential for bone regeneration, yet several challenges require improvement. While current materials enable programmable multi-stage deformations at varying humidity levels, their performance often be negatively affected in extreme humidity environment (Du et al., 2023). Also, existing systems exhibit constrained stiffness, compromising their usage in load-bearing bone defects. Last but not least, maintaining structural integrity and stable volume post-hydration is critical.

### 2.4 Piezoelectricity

Piezoelectric materials refer to certain materials that are associated with mechanical stress and the generation of electrical charges on surfaces. Generally, a piezoelectric material will generate an induced charge internally after being mechanically stressed, triggering a positive piezoelectric effect. If an electric field is subsequently applied to this material, it will cause a geometric strain, resulting in an inverse piezoelectric effect (Uchino, 2017).

Interestingly, natural bone defect healing progress, coordinately regulated by chemical, physical, and electrical signals (Pfeiffenberger et al., 2021), intrinsically leverages piezoelectricity: collagen polarization under stress generates a net negative surface charge. This attracts calcium ions into osteoblasts via voltage-gated channels, facilitating mineralization (Ahn and Grodzinsky, 2009; Khare et al., 2020). By exploiting bone’s sensitivity to these piezoelectric signals, which modulate metabolism and osteogenesis, piezoelectric materials demonstrate strong potential in bone tissue engineering (Nain et al., 2024).

Researchers explore piezoelectric materials for the complex oral-maxillofacial environment, with injectable/moldable hydrogels incorporating piezoelectric nanoparticles emerging as a central focus (Zhu et al., 2020). These composites offer excellent biocompatibility, tunable mechanics, and efficient localized electroactivity, enhancing bone repair. Innovatively, Zhou et al. incorporated dynamically covalently crosslinked piezoelectric nanoparticles into a hydrogel. This design improved material performance and significantly accelerated bone healing *in vivo*. Mechanistic studies revealed the hydrogel promotes intracellular calcium influx, continuously activating PI3K/Akt and MAPK/ERK osteogenic pathways to drive bone marrow mesenchymal stem cell differentiation (Zhou S. et al., 2024).

Successful bone repair requires inflammation control, demanding piezoelectric materials that synergistically regulate the inflammatory microenvironment. Wu et al. developed a BaTiO_3_/PDA@HA hydrogel scaffold that provides electro-immunomodulation via bioactive interfaces, promoting reparative M2 macrophage polarization via PI3K/Akt signaling to create a pro-regenerative niche (Wu et al., 2023). For inflamed environments, Ricotti et al. designed a BaTiO_3_/graphene oxide hydrogel system; its synergistic effects directly drive new bone formation in oral-maxillofacial inflammation, offering novel strategies for severe inflammatory bone defects (Ricotti et al., 2024).

Piezoelectric material applications are rapidly expanding, particularly in periosteal engineering where electrical stimulation enhances bone repair (Liu J. et al., 2023; Liu H. et al., 2023; Yue et al., 2025). Recognizing the periosteum’s critical role in early bone formation, its protection/utilization is now a key bone defect strategy (Shahabipour et al., 2023). Yue et al. developed a PVDF piezoelectric periosteal scaffold with curcumin-loaded Mg-MOF, synergistically promoting nerve repair, angiogenesis, and inflammation regulation (Yue et al., 2025). Separately, Liu et al.'s TiO_2_@PVDF nanofiber membrane (0.3 wt% TiO_2_) demonstrated markedly enhanced cell adhesion/proliferation via high surface potential, while electromechanical stimulation robustly induced early alkaline phosphatase activity–confirming electrical properties’ essential role in osteogenesis initiation (Liu J. et al., 2023).

Piezoelectric materials for oral-maxillofacial bone repair have evolved from single-component exploration to designing mechanism-driven multifunctional composites (hydrogels, fiber membranes, coated scaffolds). Future efforts must address long-term stability and precise electrical control in physiological environments, establish standardized performance comparisons, and translate findings into clinical solutions for large and infected defects.

## 3 Internal stimulation

Pathological progression closely links to altered physical/biochemical microenvironmental cues. These can act as intrinsic triggers for specific materials, triggering structural transformations that elicit biological effects on surrounding tissues. Highlighting distinctions between endogenous and exogenous stimulus strategies, this section focuses on recent advances in internal stimulus-responsive implants (Table 3).

**TABLE 3:** Types of internal stimuli, materials or methods, effects, and applications.

Stimulus	Materials or methods	Effects	Application	References
pH	Embedding pH-sensitive chemical bonds Constructing pH-sensitive materials	(1) Collapse or decomposition of the coating (2) Changes in molecular conformation (3) Breakage of chemical bonds/linkers	(1) Promoting osteoblast adhesion and altering the electrical potential of the osteogenic surface (2) Triggering the release of antibacterial and anti-inflammatory drugs	Zha et al. (2024), Sha et al. (2025)
Enzyme	Substrates corresponding to specific enzymes: (1) Matrix metalloproteinases (2) Alkaline phosphatase (3) Collagenase (4) Gingipains	Substrate degradation under the catalysis of enzymes	(1) Enzyme-triggered release of drug molecules to prevent bacterial infections or promote bone regeneration (2) Enzyme-triggered degradation and remodeling of bone repair scaffold materials	Cheng et al. (2024), Zhang et al. (2024c), Zhou et al. (2024b), Liu et al. (2025e)
Glucose	(1) Loading GOx (2) Loading phenylboronic acid groups	Enzyme-catalyzed glucose decomposition Glucose binding to phenylboronic acid groups	Enzyme-catalyzed or phenylboronic acid binding, regulating glucose concentration, alleviating inflammatory responses, triggering drug release, and promoting bone defect healing	Li et al. (2023), Liu et al. (2025d), 2025b
ROS	Constructing biomaterials whose physical properties are affected by ROS Introducing chemical bonds that react with ROS into the material: (1) Boronic ester bonds; (2) Sulfur-containing chemical bonds	Physical property changes of the material triggered by ROS Drug release under ROS-mediated biochemical reactions	Molecular release triggered by ROS response to reduce inflammatory reactions, promote osteogenesis, and regulate immune responses	Tyagi et al. (2021)

### 3.1 pH

The microenvironmental pH critically regulates tissue-engineered bone regeneration by modulating protein adsorption on artificial bone surfaces, osteogenesis-related cellular behaviors, bone matrix secretion/maturation, biomineralization, and inflammatory responses with vascular remodeling in bone defects (Liu et al., 2016; Hao et al., 2017).

Microenvironmental pH critically regulates MSCs and osteoblast proliferation (Hao et al., 2017). Acidic pretreatment (pH 6.8) enhances stem cell marker expression while improving viability and proliferation (Hazehara-Kunitomo et al., 2019). While an alkaline environment (pH 8.0–8.4) promotes initial proliferation in pre-osteoblasts, alkali-treated titanium surfaces inducing local pH elevation cause cell alkalosis and inhibit human bone marrow mesenchymal stem cell proliferation (Li et al., 2014; Galow et al., 2017). Notably, both acidic (pH 6.3/6.7) and highly alkaline (pH 8.5) conditions significantly suppress human bone marrow mesenchymal stem cell proliferation by accelerating cellular senescence, whereas physiological (pH 7.0/7.4) and mildly alkaline (pH 8.0) microenvironments optimally support cell survival and proliferation (Fliefel et al., 2016).

Oral-maxillofacial bone defects form a local acidic microenvironment due to reduced blood supply, anaerobic metabolism, and lactic acid accumulation from hematoma, infection, and inflammation (Hazehara-Kunitomo et al., 2019). Tissue-engineered bone exhibits reduced pH buffering from limited vascular ingrowth, inflammatory responses, and confined cell space, leading to acidic metabolite accumulation and heightened cellular sensitivity to pH fluctuations (Monfoulet et al., 2014; Pj and Carmeliet, 2016).

Construction strategies for pH-responsive materials comprise three main categories: functional group design, dynamic bond incorporation, and use of self-assembling peptides/nanozymes (Sha et al., 2025). Anionic polymers (e.g., polyacrylic acid) swell with cations, while cationic chitosan dissolves in acidic environments—suitable for infected bone defects (Lin et al., 2018). Dynamic bonds (e.g., hydrazone, Schiff base) enable self-healing and responses to pH/temperature (Li Z. et al., 2022). Self-assembling peptides (e.g., histidine-rich) form nanofiber gels at pH 6.0. Nanozymes like sulfur quantum dots exhibit acidic peroxidase-like activity (sterilization) and neutral catalase-like activity (bone repair) (Li Z. et al., 2022; Liu et al., 2025c).

Current pH-responsive bone repair materials primarily comprise smart polymers (e.g., polyacrylic acid, chitosan, polyline) or nanocomposite structures (e.g., Metallo phenolic networks, peptide self-assembly systems) (Bonchev and Bogovska-Gigova, 2025; Sha et al., 2025). These materials adapt to local pH changes by reversibly altering physical/chemical properties—such as swelling/contraction, degradation modulation, and drug release—enabling targeted antimicrobial delivery in the acidic microenvironment (pH 5.5–6.8) of infected oral-maxillofacial defects (Lin et al., 2025; Ma et al., 2025). During early inflammation, accelerated acidic degradation releases antimicrobial agents; as pH rises to physiological levels, degradation slows to provide sustained scaffolding for bone regeneration (Li Z. et al., 2022).

pH-responsive bone repair materials face technical limitations. Current materials require >0.5 pH unit changes for activation—insufficient for mild infections with only 0.2–0.3 unit differences (Tapponi et al., 2025). For example, metal phenolic networks coatings trigger drug release only at pH < 6.0, delaying response to early mild infections (pH 6.5–7.0) (Ali et al., 2023).

### 3.2 Enzymes

Enzyme-responsive smart materials for bone repair are a class of biomaterials that can specifically recognize changes in enzyme activity in the bone injury microenvironment and trigger their own functions (such as drug release, structural transformation, or signal transduction) accordingly (Liu X. et al., 2025).

The core principle of enzyme-responsive materials is enzyme-catalyzed reactions, and their design relies on enzyme-sensitive chemical bonds and enzyme-catalyzed signal transduction. The former involves embedding chemical bonds in the material that can be hydrolyzed or modified by specific enzymes (such as phosphate ester bonds, peptide bonds),thereby enabling the material to be triggered by specific enzymes and undergo conformational changes (Zhang M. et al., 2024; Zhou X. et al., 2024). Alkaline phosphatase, matrix metalloproteinase, collagenase, and gingipains can all serve as target enzymes (Liu et al., 2021; Liu X. et al., 2025; Xu et al., 2023; Zhou Y. et al., 2023; Zhou X. et al., 2024; Zhang M. et al., 2024).

The latter, that is, enzyme-catalyzed signal transduction, refers to the conversion of pathological signals into chemical changes recognizable by materials. These nanozymes, which have catalase, superoxide dismutase, and glutathione peroxidase enzyme-like properties, effectively reprogram the microenvironment of the mandible and treat mandibular osteoradionecrosis (Cheng et al., 2024). Compared with traditional bone repair materials, enzyme-responsive materials have the advantage that they can release drugs only in areas with high enzyme expression, thereby avoiding systemic toxicity. Enzyme activity is positively correlated with the degree of pathology, and the material can automatically adjust the release amount accordingly, avoiding excessive drug damage (Zhou X. et al., 2024).

Enzyme-responsive smart materials face clinical translation challenges primarily concerning enzyme stability. The complex *in vivo* environment can deactivate immobilized enzymes, necessitating protective strategies like nanoencapsulation (Singh et al., 2020). For materials with multiple enzymes, optimizing sequential reactions and cascade regulation remains critical. Future integration with artificial intelligence and machine learning offers potential to predict enzyme-material interactions and optimize kinetics (Kroll et al., 2023; Wang et al., 2025d).

### 3.3 Glucose

Long-term hyperglycemia can weaken the immune system of patients, leading to exacerbation of oral-maxillofacial inflammation, reduced bone repair capacity, severe loss of alveolar bone mass, poor osseointegration of dental implants, and poor repair of oral-maxillofacial bone defects (Wu et al., 2015).

The design of glucose-responsive smart materials is primarily based on two main strategies: glucose oxidase (GOx)-based system, phenylboronic acid-based system (Li et al., 2023). GOx, primarily found in human red blood cells, renal tubules, and hepatocytes, specifically catalyzes the conversion of β-D-glucose into gluconic acid and hydrogen peroxide (H_2_O_2_). Based on this principle, glucose-responsive 3D-printed scaffolds can be engineered (Liu et al., 2025b). Phenylboronic acid, as a Lewis acid containing a boron atom, has the core characteristic of being able to specifically and reversibly bind with the vicinal diol group in glucose molecules (Morariu, 2023). This unique glucose responsiveness makes it an ideal molecular tool for constructing smart delivery systems (Wang et al., 2024; Liu S. et al., 2025). Elevated blood glucose prompts phenylboronic acid groups to bind glucose preferentially. This disrupts boronate ester crosslinks, loosening the hydrogel structure. Conversely, low glucose reduces this binding, stabilizing crosslinks and maintaining a compact hydrogel to slow substance release (Wang et al., 2024).

It is worth noting that the bone tissue of patients with type 2 diabetes often has dysfunction in resistance to deformation and fracture. This decline in mechanical properties makes fracture healing even more difficult. Therefore, good mechanical properties are crucial for providing a stable microenvironment for bone tissue regeneration (Jiang et al., 2022).

Smart materials for bone repair based on GOx and phenylboronic acid groups have shown significant potential in the repair of diabetic bone defects. Although their response mechanisms are different, both can achieve dynamic regulation of the pathological microenvironment of bone defects.

### 3.4 ROS

Excessive ROS increase the apoptosis rate of osteoblasts, damage stem cell function, accelerate osteoclast differentiation and bone resorption, exacerbate inflammatory responses and vascular damage, thereby delay the healing process of bone defects (Tao et al., 2020; Ren et al., 2022).

Traditional non-degradable materials may release toxic ions in an ROS environment or hinder the ingrowth of new tissue, further delaying healing and creating a vicious cycle (Wang et al., 2025b). Whereas, the therapeutic goal of ROS-responsive materials is not simply to reduce ROS levels, but to regulate ROS levels within an appropriate range (Tyagi et al., 2021; Ren et al., 2022). Low concentrations of ROS can activate pathways such as MAPK/ERK, promoting the expression of osteogenic markers, and driving the osteogenic differentiation of MSCs. Therefore, excessive clearance of ROS may block osteogenic differentiation signals and delay bone defect healing (Mouthuy et al., 2016; Tyagi et al., 2021; Zhang Q. et al., 2024).

Interestingly, under controlled conditions, ROS can exert strong antibacterial effects (Xu et al., 2025). Recently, an emerging dynamic therapy has emerged that treats deep hypoxic infected bone defects by increasing ROS levels. This implant generates sulfate radicals (·SO_4_^−^) and ·OH in a hypoxic environment, killing bacteria through lipid peroxidation and ferroptosis mechanisms. However, the critical concentration of ROS that promotes osteogenesis still needs further investigation (Wang et al., 2025b).

ROS-responsive materials are primarily engineered around three core mechanisms: chemical bond cleavage, physicochemical property transformation, and bioactive regulation. The chemical bond cleavage strategy, utilizing ROS-sensitive bonds (e.g., thioketal, selenium-selenium bond, phenylboronic ester) that oxidatively break under high ROS levels to trigger material degradation or payload release, represents the most prevalent design approach (Li X. et al., 2022; Zhang Q. et al., 2024). Notably, bond sensitivity varies, necessitating selection based on the application context, such as chronic inflammation (Yu et al., 2022). The physicochemical property transformation​ mechanism exploits ROS-induced alterations in material state or surface characteristics, primarily categorized into hydrophobicity-to-hydrophilicity transitions (solubility switching) for controlled release and surface charge reversal to enhance cellular uptake (Zhou J. et al., 2023). Bioactive regulation extends beyond delivery, directly targeting the pathological microenvironment by incorporating ROS-scavenging antioxidants or nanozymes, or by modulating ROS levels to influence cell behavior and promote tissue repair (Yin et al., 2021; Peng et al., 2024; Zhang Q. et al., 2024).

To address complex pathological environments like infected bone defects, multi-mechanistic integration, representing a cutting-edge approach, combines strategies such as photothermal effects to accelerate ROS-sensitive bond cleavage or enzyme-responsiveness for dual-signal triggered release (Tian et al., 2022; Yu P. et al., 2023).

However, significant challenges remain: current ROS-responsive systems often exhibit linear response mechanisms, ill-suited to fluctuating ROS levels characteristic of sites like bone defects. The inherent background concentration, activity, and fluctuations of endogenous ROS often render single-mechanism responses insufficient, underscoring the urgent need for strategies responsive to multiple signals or featuring intelligent feedback control (Chen et al., 2023; Zhang Q. et al., 2024).

## 4 Conclusion

Here, we reviewed the research progress of smart materials in the field of maxillofacial bone reconstruction, discusses the characteristics of smart materials and their applications, and analyzes in detail the current status and prospects of the application of external stimulus and internal stimulus-responsive smart materials. By categorizing the types of stimuli to which smart materials respond, this paper discusses in depth the application scenarios of each type of stimuli in clinical practice and looks forward to the future development direction (Table 4). Despite promising functionalities, smart stimuli-responsive systems are still in the preclinical exploration phase, requesting intensified research efforts to bridge the gap toward clinical adoption.

**TABLE 4:** Comparison of different response strategy types.

Types	Characteristics and benefits	Existing problems	References
Photothermal	(1) Non-invasive and highly controllable (2) Significant photothermal therapeutic efficacy	(1) Limited tissue penetration depth (2) The intense photothermal effect may induce damage to adjacent normal tissues (3) Potential toxicity associated with the use of photoactivated materials	Zhang et al. (2022a), Cedillo-Servin et al. (2024), Wu et al. (2024)
Magnetic field	(1) Superior capacity for tissue penetration (2) Non-invasive and highly controllable	(1) The distribution of magnetic heat was uneven (2) Excessive localized heat may induce thermal injury to adjacent tissues	Li et al. (2020), Fragal et al. (2022)
Piezoelectricity	(1) Enhanced conductive properties (2) Significant regenerative capacity without the need for exogenous drugs or growth factors	(1) Densification, alkali volatilization, and elevated temperatures during synthesis procedures (2) Long-term biosafety and cytotoxicity profiles are yet to be fully established	Pfeiffenberger et al. (2021), Shahabipour et al. (2023)
Humidity	(1) Energy-efficient operation without external power supply (2) Reversible behavior with high cycling durability	(1) Insufficient response kinetics and material stability (2) Irreversible damage under extremely high or low humidity conditions	Zhang et al. (2022b), Hill et al. (2024)
ROS	(1) Intelligent and prompt responsiveness to environmental stimuli (2) Notable regenerative outcomes and therapeutic efficacy	(1) The limited action range and brief lifespan of ROS can significantly diminish the effectiveness of stimuli (2) This effect may also cause damage to normal cells	Mouthuy et al. (2016), Wang et al. (2025b)
pH	(1) Intelligent and swift adaptation to environmental conditions (2) Modulation of the local acidic milieu to enhance bone regenerative processes	(1) The duration of therapeutic efficacy may be insufficient to achieve optimal therapeutic outcomes (2) The prolonged acidic microenvironment may hinder subsequent bone regenerative processes	Li and Zhang (2021), Gong et al. (2024)
Enzyme	(1) Exceptional specificity towards their substrates (2) Precise and intricate process	(1) The shared substrates among closely related enzyme families may compromise specificity (Xu et al., 2023) (2) The biocompatibility and long-term cytotoxicity profiles require further assessment (3) Dysregulation of enzyme activity can influence the duration of action	Singh et al. (2020), Xu et al. (2023), Yousefiasl et al. (2023)
Glucose	(1) High Specificity with minimal interference (2) Closed-loop feedback mimicking pancreatic β-cell physiology	(1) Insufficient response kinetics and sensitivity (2) Material fatigue under repeated glucose stimulation (3) Interference from dynamic physiological microenvironments	Unruh et al. (2015), Zhang et al. (2019a), Wang et al. (2024)

**FIGURE 2:**
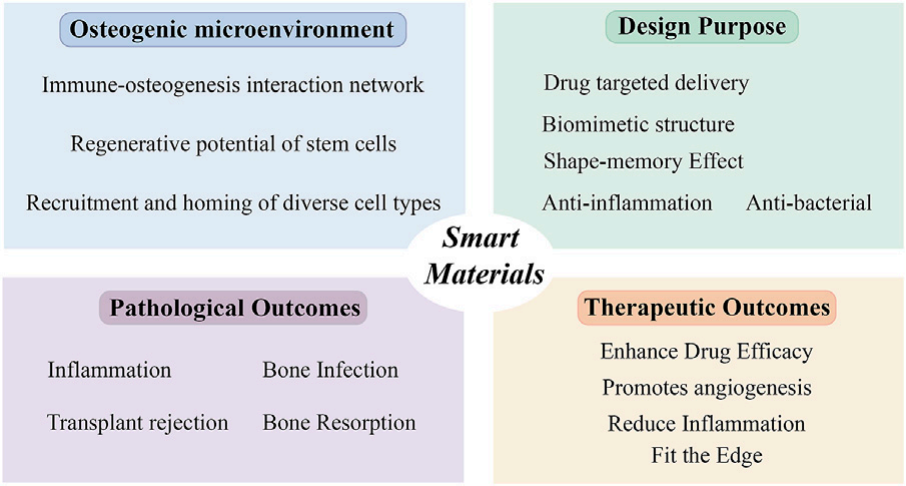
Design strategies and mechanisms of smart materials in the treatment of oral-maxillofacial bone defects.

Firstly, comprehensive biosafety assessments of these materials and their bioactive degradation byproducts are imperative. Secondly, given the prolonged regeneration timeline for bone tissue defects—particularly segmental defects requiring months of healing—coupled with lifelong remodeling processes, the long-term structural integrity and sustained responsiveness of smart bone substitutes must be rigorously validated through extended *in vivo* studies. Furthermore, osseous tissues exhibit distinct physical and mechanical characteristics compared to other clinical targets—particularly in terms of load-bearing capacity and mineralization dynamics—necessitating tailored optimization of external stimulus parameters (e.g., intensity, duration, frequency) prior to clinical deployment. Notably, internal stimuli entail patient-specific modulation due to inter-individual variability and dynamic pathophysiological progression, significantly complicating their control precision (Figure 2).
